# Revealing Associations between Diagnosis Patterns and Acupoint Prescriptions Using Medical Data Extracted from Case Reports

**DOI:** 10.3390/jcm8101663

**Published:** 2019-10-11

**Authors:** Cheol-Han Kim, Da-Eun Yoon, Ye-Seul Lee, Won-Mo Jung, Joo-Hee Kim, Younbyoung Chae

**Affiliations:** 1 Acupuncture & Meridian Science Research Center, College of Korean Medicine, Kyung Hee University, Seoul 02447, Korea; chulh92@naver.com (C.-H.K.); yde93@naver.com (D.-E.Y.); jparadise.lys@gmail.com (Y.-S.L.); croquies@naver.com (W.-M.J.); 2 Department of Anatomy and Acupoint, College of Korean Medicine, Gachon University, Seongnam 13120, Korea; 3 Department of Acupuncture and Moxibustion Medicine, College of Korean Medicine, Sangji University, Wonju 26339, Korea; jhkim714v@gmail.com

**Keywords:** acupuncture, data mining, pattern identification, network analysis

## Abstract

Objective: The optimal acupoints for a particular disease can be determined by analysis of diagnosis patterns. The objective of this study was to reveal the association between such patterns and the acupoints prescribed in clinical practice using medical data extracted from case reports. Methods: This study evaluated online virtual diagnoses made by currently practicing Korean medical doctors (N = 80). The doctors were presented with 10 case reports published in Korean medical journals and were asked to diagnose the patients and prescribe acupoints accordingly. A network analysis and the term frequency-inverse document frequency (tf-idf) method were used to analyse and quantify the relationship between diagnosis patterns and prescribed acupoints. Results: The network analysis showed that ST36, LI4, LR3, and SP6 were the most frequently used acupoints across all diagnoses. The tf-idf values showed the acupoints used for specific diseases, such as BL40 for bladder disease and LU9 for lung disease. Conclusions: The associations between diagnosis patterns and prescribed acupoints were identified using an online virtual diagnosis modality. Network and text mining analyses revealed commonly applied and disease-specific acupoints in both qualitative and quantitative terms.

## 1. Introduction

Clinical reasoning with respect to acupuncture treatment has long been considered a ‘black box’, dependent on the clinical experience and medical knowledge of individual doctors, which can be neither systemised nor quantified. The diversity in approaches is the result of the particular decision-making requirements for acupuncture treatment, which include assembling clinical data and identifying diagnosis patterns to ensure the use of appropriate acupoints [1]. Pattern identification, which is a traditional approach to diagnostic classification, can inform treatment decisions via synthesis and analysis of clinical information [2]. Different diseases (and indications) can be treated using different acupoints; individual acupoints can be applied to a wide range of indications [3,4]. Each acupoint along the different meridians showed different constellation patterns at various disease sites [5]. A review of the clinical literature on acupoint selection, and the underlying rules and patterns involved therein, showed that several different principles may apply in the treatment of a single disease [6]. Pattern identification can provide an appropriate framework for treatment selection [7,8]. Acupoints may be prescribed according to certain diagnosis patterns, where such patterns sharing common signs or symptoms can be determined by reference to commonalities in their acupoint prescriptions.

Clinical reasoning with respect to diagnosis and therapy requires comprehensive knowledge of clinical medicine and an understanding of a patient’s symptoms and signs [9]. To support clinical reasoning, digital health approaches can apply electronic processes to diagnosis and treatment, as well as specialised tools to collect and analyse the data [9]. A previous study used data mining methods to achieve an understanding of acupoint prescriptions based on diagnosis patterns, in turn based on classical medical texts [10]. Spatial patterns of representative acupoint indications have also been detailed in classical medical texts [11]. Recently, we used a bodily sensation map to visualise spatial patterns of acupoint indications and suggested a novel paradigm to explain the point specificity of acupuncture treatment [12]. In another study, the medical records of patients were collected from a physician for implementation into an artificial neural network of the diagnostic process, broadly referred to as pattern identification, using a machine learning approach [13]. Acupuncture practitioners typically engage in a series of decisions pertaining to diseases and symptoms based on clinical information collected from medical histories.

Virtual diagnoses have been employed to evaluate the clinical reasoning skill and diagnostic ability [14,15]. In accordance with previous studies, we followed the recent trend toward digital health by devising an online experiment for currently practicing doctors to collect medical information on patients. In the current study, acupuncture practitioners were provided with medical information extracted from 10 different case reports. Using the online virtual diagnoses process, acupuncture practitioners were asked to diagnose the patients and prescribe acupoints accordingly. The acupoints prescribed for each case were collected from the virtual diagnoses data completed by the participating acupuncture practitioners. Using these data, network and text mining analyses were applied to determine the relationship between diagnosis patterns and acupoint prescriptions, according to disease type.

## 2. Methods

### 2.1. Experimental Design

We conducted an online study in which physicians made virtual diagnoses. In this study, 10 cases with different diseases were presented to currently practicing Korean doctors (N = 80). The doctors were tasked with diagnosing each case and prescribing acupoints accordingly. The 10 cases were selected from among actual case reports published in Korean medical journals between 2014 and 2016. Patient information, such as symptoms, medical history, and laboratory test results, were shown. Anonymity of the participating doctors was maintained throughout the study. All procedures were conducted with the approval of the Institutional Review Board of Kyung Hee University, Seoul, Republic of Korea (KHSIRB-17-046).

**Figure 1:**
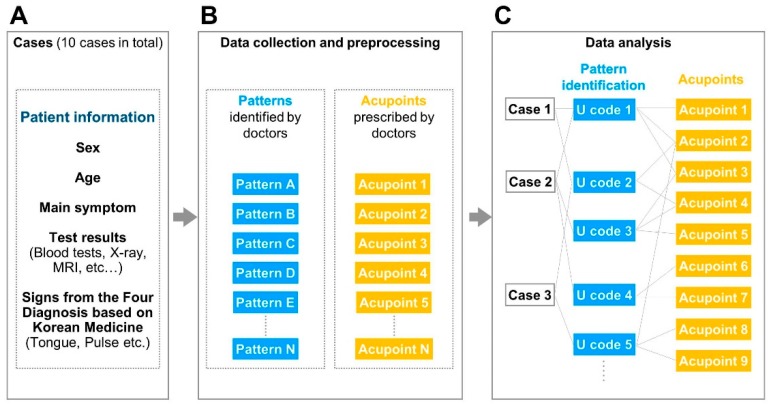
Study protocol: (**A**)**.** Ten previously published case reports were presented in this online study. Patient and disease data were provided in a single slide. The doctors were asked to diagnose the patient and prescribe acupoints accordingly. (**B**)**.** Data collection and pre-processing. The diagnosis pattern and acupoints of the doctors were obtained and pre-processed according to International Statistical Classification of Diseases and Related Health Problems (ICD)-10 codes and World Health Organization (WHO)-standardised acupoints. (**C**)**.** Data analysis. Network analysis was performed on the diagnosis patterns and prescribed acupoints, with nodes (acupoints and diagnosis patterns) and edges (correlated diagnosis patterns and acupoints). Furthermore, correlated acupoints and diagnosis patterns were extracted and subjected to term frequency-inverse document frequency (tf-idf) weighting.

Case reports not only guide personalised treatment in clinical practice, but also provide a precise means of recording diagnoses, principle treatments, outcomes, and prognoses [14]. Because case reports contain detailed clinical information about patients, they serve as valuable resources in patient-centred medicine [15]. Using previously published case studies, the presented case in the virtual diagnosis process could standardise and unify the information provided throughout the experiment. The slide for each case included information on sex, age, main symptoms, and signs associated with the diagnosis in the original case report, such as pulse and tongue condition (Figure 1A).

### 2.2. Data Collection and Pre-Processing

Using a virtual diagnosis approach, 800 medical records were collected that allowed diagnosis patterns to be identified according to Korean Medicine theory, and thus the prescription of combinations of three to five acupoints (Figure 1B). Eighty data points per case were collected for analysis in text format. Because the data were in a free-text format, without any restriction on the terminology used, the pre-processing step included unification of the diverse terminology. Pre-processing was performed using International Statistical Classification of Diseases and Related Health Problems (ICD)-10 codes, specifically U codes (U20–U99), or specialised codes specific to Korean medicine [16]. The ICD-10 codes were used because together they constitute a disease classification system incompatible with Korean medicine, thus unifying Western and traditional Korean approaches. The U codes in ICD-10 were developed to capture patterns and symptoms diagnosed only using Korean medicine. Patterns identified by the doctors in this study were matched to the most appropriate U codes. When the doctors described individual symptoms rather than the overall diagnosis pattern, an R code within ICD-10 was applied. Two senior medical students of the College of Korean Medicine provided the first-draft matching process. Finally, a Korean medical doctor with five years of training reviewed all of the diagnosis pattern U code matches. Disagreements between the researchers were discussed until a final decision was arrived upon.

Acupoint combinations were pre-processed to rule out typographical errors, and to filter out acupoints outside of the 14 meridians, as well as anatomical trigger points and auricular acupoints. After pre-processing, the Korean terms for each acupoint were converted to standard World Health Organization (WHO) terminology. Finally, the free text data were converted into .csv file format, where these files contained diagnosis pattern acupoint pairs that were then subjected to further analysis. The final pre-processed dataset contained ICD-10 disease codes and WHO standard acupoint codes; 4252 unique pairs were identified (Figure 1C). Pre-processing was conducted using an in-house script in the R software environment (ver. 3.4.2; “Short Summer” http://r-project.org/).

### 2.3. Network Analysis of Diagnosis Patterns and Prescribed Acupoints

The pre-processed data were subject to correlation analysis between diagnosis patterns and acupoint prescriptions. Thereafter, a directed network was constructed using edges pointing from each diagnosis pattern to the associated acupoints. The weights of the edges were defined according to correlations between diagnosis patterns and acupoints. The edges of co-occurrences greater than 10 were included in the network. The final network contained 13 diagnosis patterns and 29 acupoints. Eigenvector centrality was calculated to determine the acupoints that were most commonly used in acupoint prescriptions. The “igraph” R package was used to construct the network and Gephi (ver. 0.9.3) software was employed to visualize it. By studying the network patterns visually, an intuitive and comprehensive analysis was achieved.

### 2.4. Associations Between Diagnosis Patterns Acupoint Prescriptions

Correlated acupoints and diagnosis patterns within the pre-processed data were identified, to which the term frequency-inverse document frequency (tf-idf) weighting scheme was applied [17]. Within the tf-idf scheme, tf _(*t*,)_ is the frequency of term “t” in document “d”, which represents how relevant “*t*” is to “*d*”. Document frequency (df_*t*_) is the number of documents containing “t”. For tf-idf _(p,a)_ in the present study, acupoints were the “terms” and diagnosis patterns were the “documents”, such that the equation quantified the relationship between diagnosis patterns and acupoints. The relationship between acupoints and diagnosis patterns was documented only when the tf-idf value was larger than 0.1. The tf-idf results table consisted of 38 diagnosis patterns and 40 acupoints. The calculated tf-idf weights were L2-normalized by document length. The results were visualised using three-dimensional histograms or heatmaps. All text mining procedures were conducted using the “tidytext” R package. 

To determine the significance of tf-idf values, the permutation test was conducted. The permutation test is a type of statistical significance test in which the distribution of the test statistic under the null hypothesis is obtained by calculating all possible values of the test statistic under rearrangements of the labels on the observed data points. Diagnosis pattern acupoint pairs were randomly permutated, maintaining their marginal frequencies. After each permutation, tf-idf values were calculated. Permutations were repeated 10,000 times. The null distribution of the tf-idf values resulting from the permutation test was used to determine statistical significance.

## 3. Results

### 3.1. Characteristics of Diagnosis Patterns and Acupoints Based on Virtual Diagnoses

The most prevalent diagnoses are shown in Table 1. Two types of pattern were identified. The first was a “direct interpretation” linking a disease with the visceral or bodily system that it affected, such as stomach disease (U73) for gastro-oesophageal reflux disease, kidney disease (U71) for chronic prostatitis, and musculoskeletal system and connective tissue disease (U30) for intervertebral disc disorders. The second type was a “theoretical interpretation” of diseases based on the theory of Korean medicine, for example, linking the pattern of fluid and humour (U63) to benign paroxysmal positional vertigo, liver excess (U65) to menopausal climacteric symptoms and panic disorder, blood disorder (U61) to derangement of the meniscus and fibromyalgia, and qi-blood-yin-yang deficiency (U62) to diabetic neuropathy and puerperal disorder.

The most strongly correlated acupoint diagnosis pairs are listed in Table 2. Blood disorder (U61) was most frequently associated with acupoints ST36, ST35, LI4, SP6, and SP10. Qi-blood-yin-yang deficiency (U62) was most frequently associated with acupoints LR3 and KI3. Kidney disease (U71) was associated with two acupoints along the kidney meridians, KI3 and KI7, as well as with BL23, which is the Back-shu acupoint. Liver excess (U65) was associated with a liver meridian acupoint, LR3, in addition to acupoints CV17 and GV20. Finally, the pattern of fluid and humour (U63) was associated with acupoints CV12, PC6, and ST40. ST36, LI4, and SP6 were frequently prescribed across all five diagnosis patterns.

### 3.2. Network Analysis of Diagnosis Pattern Prescribed Acupoint Pairs

**Figure 2:**
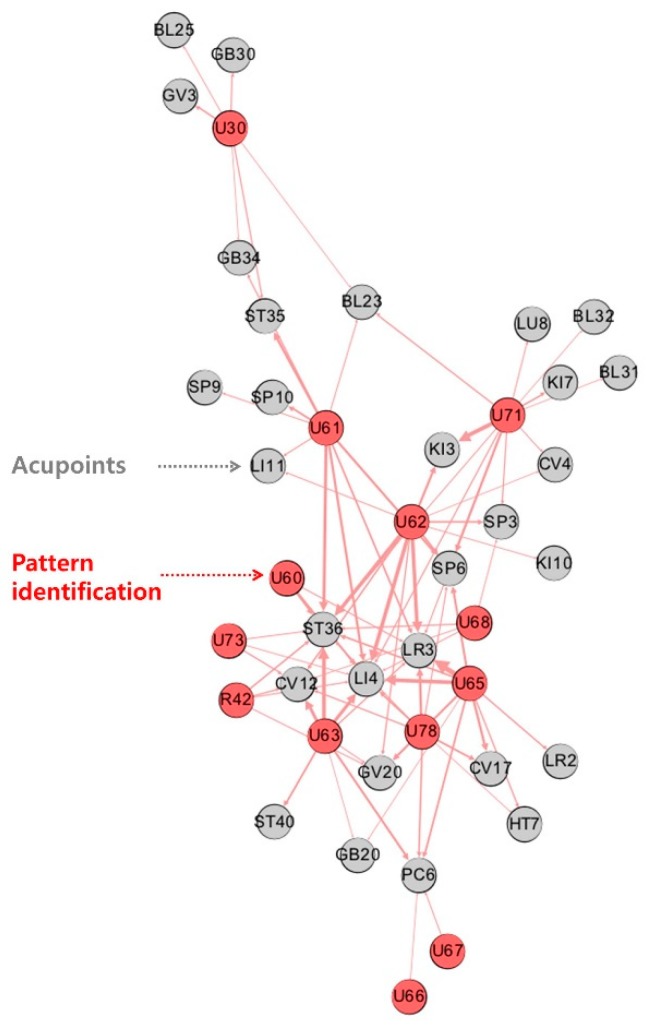
Network analysis of diagnosis patterns and prescribed acupoints. A diagnosis pattern -acupoint network was generated featuring nodes (acupoints) and edges (pairs of correlated acupoints). R42 (vertigo), U30 (diseases of the musculoskeletal system and connective tissue), U60 (qi deficiency), U61 (blood disorder), U62 (qi-blood-yin-yang deficiency), U63 (pattern of fluid and humour), U65 (liver excess), U66 (heart deficiency), U67 (heart excess pattern), U68 (spleen disease), U71 (kidney disease), and U73 (stomach disease) were included. Red and grey nodes represent diseases and acupoints, respectively. Nodes with higher eigenvector centrality are located in the centre of the network. The thickness of the edge is proportional to the frequency of correlations between linked nodes.

The final network included 13 diagnoses and 29 acupoints (Figure 2). Two features of the network analysis were examined. First, clusters of diagnoses and acupoints were distinguishable. For example, acupoints SP10 and ST35 were specifically clustered with blood disorder (U61); GV3 and BL25 with diseases of the musculoskeletal system and connective tissue (U30); and LU8, BL31, BL32, and KI7 with the kidney disease pattern (U71). Other diagnoses shared acupoints. For example, heart deficiency (U66) and heart excess pattern (U67) shared acupoint PC6. Second, acupoints showing the highest eigenvector centrality (ST36: 1.00; LI4: 1.00; LR3: 0.79; SP6: 0.61; CV12: 0.59) were those that were shared by several diagnoses. Specifically, ST36 and LI4 had an eigenvector centrality of 1.00, implying that they were prescribed at least once for every diagnosis pattern.

### 3.3. Associations Between Diagnosis Patterns and Prescribed Acupoints

**Figure 3:**
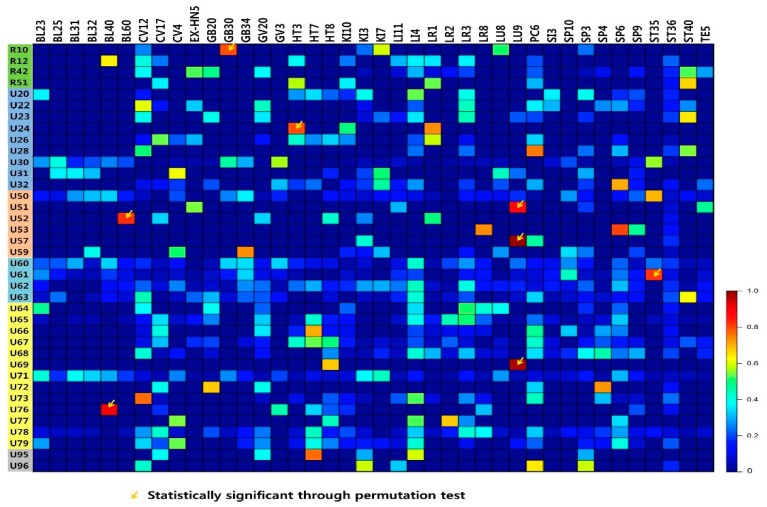
Associations between diagnosis patterns and acupoints. The significantly associated diagnosis patterns and acupoint codes were as follows: BL40 with U76; BL60 with U52; GB30 with R10; HT3 with U24; LU9 with U51, U57, and U69; and ST35 with U61. Acupoints are on the x-axis. On the y-axis, 38 diagnosis patterns are represented; the corresponding ICD-10 codes are shown as different-coloured boxes (green: symptom-based codes; blue: diseases defined in Korean medicine; orange: pattern of six meridians and external contractions; purple: qi-blood-yin-yang deficiency -Fluid-Humour; yellow: visceral system and bowel-related; and grey: Sasang constitution).

The tf-idf values show the acupoint prescriptions by diagnosis. The tf-idf values for 40 acupoints and 38 diagnosis patterns are shown on a heatmap in Figure 3. Acupoints prescribed for a specific disease, such as ST35 for blood disorder (U61) and GV3 and BL25 for diseases of the musculoskeletal system and connective tissue (U30), had high tf-idf values. Acupoints that were omitted from the network analysis owing to low prescription frequency, but were nonetheless associated with specific diseases, also had high tf-idf values.

The permutation test showed that the tf-idf values of acupoint BL40 for bladder disease (U76); BL60 for greater yang heat (U52); GB30 for pelvic pain (R10); HT3 for numbness and insensitivity (U24); LU9 for yang disease (U51), yin disease (U57), and lung disease (U69); and ST35 for blood disorder (U61) reached statistical significance; this indicates that these acupoints are specifically prescribed for these diagnosis patterns.

## 4. Discussion

This study investigated acupoint prescriptions by diagnosis patterns using network and text mining analyses. The data used in this study were collected from medical records obtained via virtual diagnoses, which were performed by currently practicing doctors. Two types of pattern were identified: the direct interpretation pattern, linking a disease with the visceral system; and theoretical interpretation of diseases based on Korean medicine principles. Some acupoints were related to specific diseases, while others were common to several diseases. The tf-idf values showed the acupoints specifically associated with certain diseases, such as GB30 for pelvic pain and LU9 for lung deficiency. In addition, the permutation test was applied to determine acupoints having statistically significant associations with diagnosis patterns. Network and text mining analyses qualitatively and quantitatively determined the acupoints specific to certain diseases, and those shared by several diseases.

Among the different diseases in this study, some were directly related to the visceral or anatomical system and there were others in which the characteristics of the disease were interpreted according to pattern identifications. Examples of the first type, which are easier to understand, include stomach disease (U73), corresponding to gastro-oesophageal reflux disease; kidney disease (U71), corresponding to chronic prostatitis; and diseases of the musculoskeletal system and connective tissue (U30), corresponding to intervertebral disc disorders. An example of the second disease type is liver excess (U65), corresponding to menopausal climacteric symptoms and panic disorder. A previous study described the role of the liver in emotional states by reference to the classical medical text, DongUiBoGam [18]. Interestingly, panic disorder and menopausal climacteric symptoms identified by the doctors in this study were most frequently linked to liver excess, which accords with the link between emotional disorders and the visceral system assumed in Korean medicine. The association between blood disorder (U61) and derangement of the meniscus and fibromyalgia, and that between qi-blood-yin-yang deficiency (U62), diabetic neuropathy, and puerperal disorder, showed that the underlying cause of a disease, as understood in Korean medicine, rather than the disease itself, is often crucial when prescribing therapies.

Network and text mining analyses comprehensively characterised acupuncture prescriptions that were associated with specific diseases, and those that were shared by several diseases, both qualitatively and quantitatively. The characteristic of the prescription of these acupoints shows a systematic body of empirical knowledge of the interconnections that underlie pathology in a particular disease [5,6]. For example, acupoint GB30 is located in the low back and hip area, which explains its use to treat pelvic pain. On the other hand, LU9 may be used to treat lung deficiency because of its meridian characteristic. Previous studies have described acupoints and meridians in spatial terms based on classical medical texts [11]. The results of this study are consistent with the local indications of the acupoints, as well as the expanded implication of systemic indications, albeit shown by data collected in a different way, namely by currently practicing doctors. Acupoints ST36, LI4, and LR3 were frequently prescribed in the majority of diseases. These acupoints also had the highest eigenvector centrality values in the network analysis (eigenvector > 0.7), showing that they were common among all of the diagnosis patterns. A previous study also that reported acupoints such as ST36 were associated with numerous disorders and diagnosis patterns [10]. However, it is unclear whether such acupoints are merely habitually selected by doctors, or are actually the most effective.

Data mining methodologies, including network analysis and text mining, have been widely used for the evaluation of diagnosis patterns and syndrome differentiation [19]. Data from clinical trials have been analysed to identify associations between certain patterns and diagnoses in Western medicine [20]. Data mining methods including network analysis [21], support vector machine classification [22], multi-label learning [23,24], random forest classification [25], artificial neural networks [13,26], and deep learning [27] have also been applied to the clinical databases of traditional Chinese and Korean medicine, to identify and predict diagnosis patterns by disease category, or in the context of a single doctor. Although data heterogeneity and a low prediction rate might limit the application of these analysis methods to clinical practice, or to the standardisation of diagnosis patterns at the individual disease level, studies have nevertheless provided insight into the potential utility of data mining for diagnosis pattern-related and herbal medicine prescription-related research. Through use of network and text mining analyses, this study supported previous findings by visualising associations between diagnosis patterns and acupoints in the context of decision-making by doctors.

This study had certain limitations. First, the data processing was done manually and the researchers determined diagnosis patterns based on free-text data. Such limitations have been present in numerous studies and remain to be fully resolved. Previous studies used classical medical texts, the clinical literature, or medical records databases to understand associations between diagnosis patterns and acupoint prescriptions. Although the results have been instructive, ethical issues, heterogeneity of data, or a lack of clinical applicability have been persistent problems. By including recent case reports, this study attempted to circumvent the problems of heterogeneous data and lack of clinical applicability. In addition, it showed that medical decision-making pertaining to diagnosis, therapy, and clinical reasoning can be systemised through analysis of previously published case reports. Finally, most of the doctors who took part in our study had less than 10 years experiences (81.3%). The diagnosis skills on pattern identification might be different based on the experience of doctors. It will be interesting to compare the diagnosis skills on pattern identification considering a variety of experience of practitioners in future study.

## 5. Conclusions

In conclusion, this online study explored the association between diagnosis patterns and acupoint prescriptions via a virtual diagnosis modality. Case reports were used such that the information provided to the doctors was in a standardised format, thereby providing a concrete basis for the patterns and acupoints decided on by the different doctors. Network and text mining analyses comprehensively showed the diagnosis patterns, as well as acupoint prescriptions that were specific to a particular disease or showed an association with several different diseases.

## Figures and Tables

**Table 1:** The most prevalent diagnoses.

Case	Diagnosis		%
1	U63	Fluid and humour	40.6
2	U73	Stomach disease	28.5
3	U65	Liver excess	27.8
4	U61	Blood disorder	40.3
5	U62	Qi-blood-yin-yang deficiency	23.8
6	U71	Kidney disease	38.6
7	U65	Liver excess	30.7
8	U30	Diseases of the musculoskeletal system and connective tissue	33.8
9	U61	Blood disorder	19.9
10	U62	Qi-blood-yin-yang deficiency	39.7

**Table 2:** The top five most frequent diagnoses and prescribed acupoints.

Diagnosis Pattern Code Frequency (n)	Acupoints Frequency (%)				
**U61**	**ST36**	**ST35**	**LI4**	**SP6**	**SP10**
460	32 (7.0%)	27 (5.9%)	26 (5.7%)	25 (5.4%)	21 (4.6%)
**U62**	**LI4**	**ST36**	**LR3**	**KI3**	**SP6**
432	38 (8.8%)	37 (8.6%)	36 (8.3%)	25 (5.8%)	21 (4.9%)
**U71**	**KI3**	**SP6**	**KI7**	**BL23**	**LI4**
424	40 (9.4%)	25 (5.9%)	18 (4.2%)	16 (3.8%)	15 (3.5%)
**U65**	**LR3**	**LI4**	**CV17**	**GV20**	**SP6**
397	49 (12.3%)	38 (9.6%)	26 (6.5%)	24 (6.0%)	24 (6.0%)
**U63**	**ST36**	**LI4**	**CV12**	**PC6**	**ST40**
378	39 (10.3%)	33 (8.7%)	32 (8.5%)	24 (6.3%)	21 (5.6%)

## Abbreviations

ICD-10: International Statistical Classification of Diseases and Related Health Problems-10 codes; WHO: World Health Organization; tf-idf: term frequency-inverse document frequency.
